# Role of Family Milieu in Tobacco Addiction: A Study in a Tertiary-care Institution in India

**DOI:** 10.3329/jhpn.v31i1.14757

**Published:** 2013-03

**Authors:** Shridhar Dwivedi, Amitesh Aggarwal, Nishant Singh, Sourabh Aggarwal, Vishal Sharma

**Affiliations:** ^1^Department of Medicine/Preventive Cardiology, Hamdard Institute of Medical Sciences and Research, Jamia Hamdard (Hamdard University), New Delhi 110 062, India;; ^2^Department of Preventive Cardiology/Medicine, University College of Medical Sciences (University of Delhi) and GTB Hospital, Delhi, India

**Keywords:** Addiction, Family, Non-communicable disease, Public health, Tobacco, India

## Abstract

Use of tobacco is singularly responsible for most cases of cancer and coronary artery disease (CAD). Efforts to stop tobacco-use need to be guided by social circumstances. It is believed that family milieu may play a role in tobacco addiction. We studied the prevalence and pattern of tobacco-use in families of 50 consecutive tobacco-user patients who presented to a tobacco-cessation clinic and compared with age- and gender-matched controls (non-users of tobacco). The tobacco-use rates were significantly higher in the family of patients with tobacco-use compared to the control group. We conclude that problems of tobacco-use are not related to individual phenomenon, and efforts for control of tobacco addiction must be focused on entire family.

## INTRODUCTION

Worldwide, tobacco-use continues to be one of the leading causes of preventable death and has been estimated to kill more than five million people annually (1). In recent times, the epidemic of tobacco-use has shifted from developed to developing countries (2). According to estimates, 10 million people will die from tobacco-use per year by 2030, with 70% of these deaths occurring in developing countries (1). India, a major developing country, accounts for one-sixth of the tobacco-related illnesses worldwide and is estimated to face an exponential increase in tobacco-related mortality from 1.4% of all deaths in 1990 to 13.3% in 2020 (3). To reduce the burden of morbidity and mortality due to tobacco-use in population, it is essential that effective policies for tobacco control be implemented.

To formulate an effective strategy for tobacco control, it is very essential to know how the habit of tobacco-use gets initiated so that this can be prevented at the initial stages. One of the essential questions is whether one learns tobacco consumption at home or outside home. We hypothesize that tobacco consumption is primarily learnt at home. Therefore, for tobacco control to be efficient, the home environment should be the primary target. No earlier study has focussed on this issue in this geographical area. With this background, we studied the pattern of tobacco-use in families of tobacco-users and non-users and their potential influence on the habit of tobacco consumption in siblings and their children.

## MATERIALS AND METHODS

This prospective cohort study was performed in a tertiary-care hospital in Delhi. The study was initiated in November 2010 and included new patients who were registered in a tobacco-cessation clinic run for coronary artery disease patients with history of smoking. Fifty consecutive patients over the next three months were enrolled. The approval for the study was obtained from the Ethics Committee of the hospital. The patients with coronary artery disease, with history of tobacco-use (smoking and/or tobacco-chewing), were enrolled in Group I. Prevalence of the use of tobacco among the parents, siblings, and children were found out by creating pedigree profile of such patients. Another 50 age- and gender-matched controls who did not use tobacco in any form were enrolled in Group II. These included healthy relatives of patients visiting outpatients department and the hospital staff. They were similarly evaluated for history of the use of tobacco in family by creating pedigree profiles for these patients since pedigree profiles provide detailed information about smoking, psychosocial factors (conflicts in family/stress), family history of premature cardiovascular disease, hypertension, and diabetes. Pedigree assessment gives us an opportunity for early lifestyle intervention in young asymptomatic siblings (4). Prevalence of the use of tobacco among both groups was compared and analyzed using SPSS (version 17.1) and any statistically-significant difference noted. For the purpose of the study, p values <0.05 were considered statistically significant.

## RESULTS

There were 50 patients with CAD in Group I and 50 age- and gender-matched healthy controls in Group II. Both cases and controls had an average age of 44.2±16.3 years. There were 12 females (24%) and 38 males (76%) among the cases and 12 females (24%) and 38 males (76%) among the controls.

In Group I, both mother and father were using tobacco in the case of 12 tobacco-users with CAD. Among tobacco-users, 42 patients (84%) had history of tobacco-use by fathers and 14 patients (28%) had history of tobacco-use by mothers compared to only 1 subject (2%) with history of tobacco-use by fathers and none (0%) by mothers of the non-users (Table). The use of tobacco among parents in Group I was higher than in Group II, and it was statistically significant with p<0.001.

The siblings of 35 patients (70%) in Group I used tobacco in multiple forms compared to 0% in Group II controls. Also, 12 children (24%) of patients in Group I used tobacco compared to 0% children of controls in Group II. There was higher use of tobacco in different forms in children and siblings of Group I patients compared to healthy controls in Group II, and the difference was statistically significant (p<0.001). Prevalence of the use of tobacco among family members of cases and controls is shown in the table.

**T1able.** Profile of the use of tobacco among family members of cases and controls

Parameter	Tobacco-user indexed cases (n=50)	Healthy controls (n=50)	p value
Individuals having tobacco-user father	42	1	<0.001
% of individuals having father using tobacco	84%	2%	
Individuals having tobacco-user mother	14	0	<0.001
% of individuals having mother using tobacco	28%	0	
Individuals having tobacco-user sibling(s)	35	0	<0.001
% of individuals having sibling(s) using tobacco	70%	0	
Individuals having tobacco-user children	12	0	<0.001
% of individuals having children using tobacco	24%	0	

Among the tobacco-user CAD patients in Group I, 26 (52%) used *bidi*, 9 patients (18%) used *guthka*, 16 patients (32%) used other forms of oral intake of tobacco, 6 patients (12%) used cigarette, and 7 patients (14%) were users of poly-tobaccos.

## DISCUSSION

The present study reveals that members of tobacco-user families were more likely to be using tobacco compared to those of families not using tobacco, and this use of tobacco in the families of tobacco-users was statistically significant (p<0.001). Previous studies analyzing the impact of the use of tobacco in family on acquiring this habit have also shown similar results (5,6). The importance of this study lies in the fact that it is the first study known to us analyzing the family influence of smoking in this geographical area. A very recent study by D'souza *et al*. showed that, up to 79% of tobacco-users who presented to a smoking-cessation clinic reported a family member using tobacco (6). Shamsuddin *et al*. further showed that effect of the father's smoking habit on the child's current smoking habit is significant, even after controlling for other familial and non-familial factors, including parental supervision, academic performance, reported influence of cigarette advertisement, and having friends who smoked (5). Our study also showed that, among tobacco-users, 84% reportedly had history of tobacco-use by fathers.

The first lessons of smoking and tobacco consumption are usually learnt at home from a parent or a grown up sibling, if the habit is acquired at an early age before 18 years (7). During their formative phase, kids who see their parents, elders, and siblings to smoke or chew tobacco become inquisitive, and their minds get primed and conditioned to initiate smoking, at quite a young age. The most dominant role is played by mother in early enunciation of smoking or tobacco-use by offspring. The logic behind such hypothesis is that, if a mother has been smoking, the foetus passively inhales nicotine and carbon monoxide through placenta while in mother's womb (8). Soon after delivery, the baby is again subjected to passive smoking while in the lap of mother during breastfeeding or otherwise. The visual impact on brain during immediate neonatal period, i.e. seeing one's own mother smoking, is something which has not been studied extensively but one can safely surmise that such children will emulate smoking much earlier than those whose mothers have not been smoking during pregnancy and subsequent period. It has also been observed that the brain of the foetus whose mother is a smoker gets primed for nicotine craving and smoking during their adolescence (9,10). The resultant effect is that most offspring of such parents would adopt smoking early and be the victims of premature CAD and other tobacco-related diseases. The combined ill-effect of maternal smoking/passive smoking is not only epigenetic and priming to the foetus but also adventurous and fascinating, finally resulting in addictive habit as early as 6 or 7 years of age.

Once habit of tobacco consumption is acquired at young age, it is difficult to get rid of it because of its addictive nature, even if someone gets a disease due to its ill-effects. One not only inherits the habit of smoking from parents/elders but also passes on the baton to the next generation. This signifies that smoking or chewing tobacco is not an individual phenomenon but rather a familial phenomenon, and so, approaches targeting families on the whole will tend to be more effective while formulating strategies for the control of tobacco-use.

### Limitations

A major limitation of our study is that the sample-size is small. Also, the report includes family history from patients only and does not include healthy smokers.

### Conclusions

Our study indicates that, in the families of tobacco-user CAD patients, consumption of tobacco by parents and siblings is higher compared to families not using tobacco. There is also a greater prevalence of tobacco-use among children of the tobacco-user families compared to non-user families. This has great public-health implications in tobacco-control programme, warranting intensive health education focused on entire family where elders are tobacco-users. One not only inherits the habit of smoking from parents/elders but also passes on the baton to the next generation.

A large-scale study involving data from a large population can more significantly guide formulation of policies for effective prevention of the use of tobacco.
